# The significance of admission blood lactate and fibrinogen in pediatric traumatic brain injury: a single-center clinical study

**DOI:** 10.1007/s00381-023-06257-9

**Published:** 2023-12-26

**Authors:** Kun-yuan Zhang, Pei-long Li, Peng Yan, Cheng-jian Qin, Hao He, Chang-pin Liao

**Affiliations:** 1 Department of Neurosurgery, Second People’s Hospital of Pingchang, Pingchang, Sichuan P.R. China; 2 Kunming Children’s Hospital, Children’s Hospital Affiliated to Kunming Medical University, Kunming Medical University, Kunming, P.R. China; 3 Department of Neurosurgery, Affiliated Hospital of Youjiang Medical University for Nationalities, Baise, Guangxi P.R. China; 4 Department of Neurosurgery, People’s Hospital of Baise, No. 8, Chengxiang Street, Youjiang District, Baise, Guangxi P.R. China

**Keywords:** Blood lactate, Fibrinogen, Traumatic brain injury, Children

## Abstract

**Background:**

Traumatic brain injury (TBI) is a significant cause of morbidity and mortality in pediatric patients, leading to long-term physical, cognitive, and psychological impairments. Blood lactate and fibrinogen levels have emerged as potential biomarkers associated with tissue hypoperfusion and coagulation dysfunction, respectively. However, limited research has specifically focused on the significance of these biomarkers in pediatric TBI. This study aimed to investigate the clinical significance of blood lactate and fibrinogen levels upon admission in pediatric patients with traumatic brain injury.

**Methods:**

The medical records of 80 children with a traumatic brain injury who were admitted from January 2017 to January 2021 were retrospectively analyzed. The two groups were compared according to whether the blood lactate in the admission arterial blood gas increased and the fibrinogen content in the coagulation function decreased. The clinical data of the children in the two groups were different, and then they were divided into a good prognosis group and a poor prognosis group according to the GOS prognostic score, and the differences in the clinical indicators of the two groups were compared.

**Results:**

Among the 80 patients, 33 had elevated blood lactate levels, 34 had decreased fibrinogen levels, and 29 had an unfavorable outcome (GOS < 4). Compared to the normal blood lactate group, there were no statistically significant differences in age, sex ratio, or platelet count in the elevated blood lactate group (*P* > 0.05). However, the elevated blood lactate group had lower Glasgow Coma Scale (GCS) scores upon admission, higher blood lactate levels, lower fibrinogen levels, longer hospital stay, lower GOS scores, and a higher proportion of GOS < 4 (*P* < 0.05). Compared to the normal fibrinogen group, there were no statistically significant differences in age, sex ratio, or platelet count in the decreased fibrinogen group (*P* > 0.05). However, the decreased fibrinogen group had lower GCS scores upon admission, higher blood lactate levels, lower fibrinogen levels, longer hospital stays, lower GOS scores, and a higher proportion of GOS < 4 (*P* < 0.05). Compared to the favorable outcome group, there were no statistically significant differences in age, sex ratio, or platelet count in the unfavorable outcome group (*P* > 0.05). However, the unfavorable outcome group had lower GCS scores upon admission, higher blood lactate levels, lower fibrinogen levels, longer hospital stays, a higher incidence of pulmonary infection, a higher incidence of stress ulcers, and lower GOS scores (*P* < 0.05).

**Conclusion:**

The levels of blood lactate and fibrinogen may represent the severity of children with traumatic brain injury and may be risk factors for poor prognosis of children with traumatic brain injury.

## Introduction

Trauma is reported as the leading cause of mortality among children in developed countries, with TBI accounting for a significant proportion of these fatal traumatic conditions [1]. Children, due to their ongoing organ system development, undergo complex and varied pathological and physiological changes following TBI, which may lead to various complications and long-term sequelae. These can include psychiatric and cognitive abnormalities, seizures, speech and motor function impairments, and in some cases, these complications can have lifelong implications, imposing significant pressure on the affected children, their families, and society [2, 3].

## Materials and methods

### Clinical data

We collected medical records of 80 pediatric patients with TBI who were admitted to the Department of Neurosurgery from January 2017 to January 2021. The inclusion criteria were as follows [3–6]: (1) Age ≤ 18 years with a documented history of trauma; (2) Confirmed diagnosis of TBI based on head CT scan; and (3) Hospital admission within 24 h of injury. The exclusion criteria were as follows: (1) Age > 18 years or unclear history of trauma; (2) Admission to the hospital more than 24 h after the injury; and (3) The presence of multiple injuries, complex injuries, or concomitant significant organ system diseases involving the heart, liver, lungs, kidneys, etc.

### Methods

#### Data collection

Clinical data of the pediatric patients were collected by retrieving records from the hospital’s medical record system. The following information was recorded: baseline characteristics upon admission, including demographic indicators, time of injury, mechanism of injury, duration from injury to hospital admission, and GCS score upon admission; relevant examination results, including blood lactate level, coagulation function, platelet count, and CT scan findings; and the progression of the patients’ condition and outcome indicators, including surgical treatment, intracranial infection, pulmonary infection, stress ulcers, and GOS score at 3 months (GOS ≥ 4 indicated a favorable prognosis, GOS < 4 indicated an unfavorable prognosis).

#### Specimen collection methods and criteria

Arterial and venous blood samples were collected within 30 min after admission. Arterial blood samples were used for arterial blood gas analysis, while venous blood samples were used for fibrinogen detection. Blood lactate levels were measured using the arterial blood lactate obtained from arterial blood gas analysis. The measurements were performed using a blood gas analyzer and corresponding reagent kit produced by Shenzhen Mindray Bio-Medical Electronics Co., Ltd., using dry chemistry or the alternating current impedance method for analysis. Fibrinogen levels were determined using the ACL TOP700 fully automated coagulation analyzer and fibrinogen assay kit (clotting method) for venous blood fibrinogen measurement. A blood lactate level greater than 2.2 mmol/L was considered elevated, while a blood lactate level of 2.2 mmol/L or lower was considered normal. A fibrinogen level less than 2.0 g/L was considered decreased, while a fibrinogen level between 2.0 g/L and 4.0 g/L was considered normal.

#### Statistical analysis

The clinical data and analyzed data were recorded using Microsoft 2016 and analyzed using SPSS 23.0. Normally distributed continuous data are presented as the mean ± standard deviation (*X ± S*), and the comparison of the means between two groups were performed using a t test. Nonnormally distributed data were presented as the median and interquartile range (*M [P25, P75]*). Categorical data are presented as the relative numbers or rates, and the chi-square test was used for comparison.

## Results

### Comparison of the clinical data between the elevated blood lactate group and normal blood lactate group

Among the 80 patients, 33 had elevated blood lactate levels. There were no significant differences between the elevated blood lactate group and the normal blood lactate group in terms of age, sex ratio, and platelet count (*P* > 0.05, Table 1). However, the elevated blood lactate group had lower GCS scores upon admission, higher blood lactate levels, lower fibrinogen levels, longer hospital stays, lower GOS scores, and a higher proportion of patients with GOS < 4 (*P* < 0.05, Table 1).

**Table 1:** Comparison of clinical characteristics between the elevated blood lactate group and the normal blood lactate group

clinical data	elevated blood lactate group(n = 33)	normal blood lactate group(n = 47)	*t/c2*	*P* value
age (years)	8.55 ± 1.70	8.79 ± 1.74	0.617	0.54
gender (male/female)	20/13	27/20	0.080	0.78
blood lactate level	2.55 ± 0.31	1.66 ± 0.33	12.200	<0.001*
platelet count	197.79 ± 54.63	199.28 ± 55.59	0.119	0.91
fibrinogen level	1.77 ± 0.67	2.32 ± 0.63	3.763	<0.001*
GCS score at admission	6.61 ± 1.46	12.34 ± 2.00	14.035	<0.001*
Length of stay	28.73 ± 8.89	16.36 ± 9.20	5.998	<0.001*
GOS score	2.94 ± 0.788	4.49 ± 0.66	9.58	<0.001*
poor prognosis	25	4	37.938	<0.001*
good prognosis	8	43		

Note: * indicates statistically significant differences (*P* < 0.05)

### Comparison of the clinical data between the fibrinogen decreased group and fibrinogen normal group

Among the 80 patients, 34 had decreased fibrinogen levels. There were no significant differences between the fibrinogen-decreased group and the fibrinogen-normal group in terms of age, sex ratio, and platelet count (*P* > 0.05, Table 2). However, the fibrinogen-decreased group had lower GCS scores upon admission, higher blood lactate levels, lower fibrinogen levels, longer hospital stays, lower GOS scores, and a higher proportion of patients with GOS < 4 (*P* < 0.05, Table 2).

**Table 2:** Comparison of clinical characteristics between the decreased fibrinogen level group and the normal fibrinogen level group

clinical data	decreased fibrinogen level group(n = 34)	normal fibrinogen level group(n = 46)	*t/c2*	*P* value
age (years)	8.53 ± 1.83	8.80 ± 1.64	0.705	0.48
gender (male/female)	20/14	27/19	0.000	0.990
fibrinogen level	1.49 ± 0.30	2.53 ± 0.56	9.868	<0.001*
blood lactate level	2.42 ± 0.37	1.74 ± 0.47	6.892	<0.001*
platelet Count	199.64 ± 50.46	197.93 ± 58.43	0.137	0.89
GCS score at admission	7.38 ± 2.36	11.89 ± 2.62	7.934	<0.001*
Length of stay	28.32 ± 8.87	16.39 ± 9.44	5.732	<0.001*
GOS score	3.03 ± 0.80	4.46 ± 0.75	8.183	<0.001*
poor prognosis	24	5	30.170	<0.001*
good prognosis	10	41		

Note: * indicates statistically significant differences (*P* < 0.05)

### Comparison of the clinical data between the poor prognosis group and good prognosis group

Among the 80 patients, 29 had a poor prognosis (GOS score < 4). There were no significant differences between the poor prognosis group and the good prognosis group in terms of age, sex ratio, and platelet count (*P* > 0.05, Table 3). However, the poor prognosis group had lower GCS scores upon admission, higher blood lactate levels, lower fibrinogen levels, longer hospital stays, a higher proportion of patients with pulmonary infections, a higher incidence of stress ulcers, and lower GOS scores (*P* < 0.05, Table 3).

**Table 3:** Comparison of clinical characteristics between the poor prognosis group and the good prognosis group

clinical data	poor prognosis (n = 29)	good prognosis (n = 51)	*t/c2*	*P* value
age (years)	8.31 ± 1.67	8.90 ± 1.72	1.492	0.140
gender (male/female)	16/13	31/20	0.240	0.624
fibrinogen level	1.67 ± 0.68	2.33 ± 0.60	4.517	<0.001*
blood lactate level	2.52 ± 0.36	1.74 ± 0.42	8.348	<0.001*
platelet Count	200.41 ± 53.41	197.67 ± 56.16	0.214	0.831
GCS score at admission	6.79 ± 1.93	11.78 ± 2.55	9.147	<0.001*
Length of stay	29.93 ± 8.64	16.65 ± 9.00	6.443	<0.001*
pulmonary infection	16	6	17.472	<0.001*
stress ulcer	8	2	9.466	0.002
GOS score	2.66 ± 0.55	4.53 ± 0.50	15.437	<0.001*

Note: * indicates statistically significant differences (*P* < 0.05)

## Discussion

In recent years, there has been a significant improvement in the understanding of the pathology of TBI. Additionally, TBI is a leading cause of disability and death in children. The series of injuries that occur following pediatric TBI, including scalp injuries, skull fractures, and brain tissue damage, are similar to those seen in adults. However, due to the immaturity of various organ systems in children, if effective treatment is not provided following TBI, it may have lifelong detrimental effects in children. Statistics have shown that over 470,000 children aged 0–4 years suffered from TBI, with more than 30,000 requiring hospitalization and over 2,000 deaths resulting from TBI, in the United States [7, 8].

Blood lactate, a product of anaerobic glycolysis in the body, is normally processed by the liver to maintain stable levels. When lactate production exceeds the liver’s processing capacity, lactate accumulation occurs in the body. Therefore, the level of lactate in the body can reflect glucose metabolism, tissue perfusion, and other conditions. There is evidence that lactate levels can reflect the severity and prognosis of neurological diseases such as aneurysmal subarachnoid hemorrhage [9].

Our study divided the patients into an elevated blood lactate group and a normal blood lactate group. We found that the patients in the elevated blood lactate group had lower GCS scores upon admission, higher blood lactate levels, lower fibrinogen levels, longer hospital stays, lower GOS scores, and a higher proportion of patients with GOS < 4. The same results were also confirmed in Wang’s study [10]. A study conducted at the Nanjing General Hospital of Nanjing Military Command showed that blood lactate levels were correlated with heart rate and systolic blood pressure, which are classical prognostic indicators for shock patients. The study also found that arterial blood lactate levels in patients who died from traumatic brain injury were significantly higher than those in nondeath patients.

Moreover, in our findings, the patients were classified into good prognosis and poor prognosis groups, and we found that the blood lactate levels in the good prognosis group were significantly lower than those in the poor prognosis group (*P* < 0.001). The same results were also confirmed in Svedung’s study [11]. In a case analysis conducted at the Neurosurgery Department of Qinghai Provincial People’s Hospital, strict control of each step was implemented, and for the first time, the volume of cerebral contusion and the average CT value in the edema area were included in the model. It was found that the ratio of glucose to lactate in cerebrospinal fluid was an independent risk factor for poor prognosis in cerebral contusion [12]. A study [13] has shown that in patients with spontaneous intracerebral hemorrhage, fibrinogen levels are an independent predictor of hematoma expansion. In our study, based on whether fibrinogen levels were decreased, we found that the patients in the decreased fibrinogen group had lower GCS scores upon admission, higher blood lactate levels, lower fibrinogen levels, longer hospital stays, lower GOS scores, and a higher proportion of patients with GOS < 4 (*P* < 0.05). In Zhang’s study [14], plasma fibrinogen levels were found to be an independent factor influencing intracranial hematoma volume in patients with acute traumatic brain injury (*r = -3.40, P < 0.001*). Moreover, the group of patients with poor prognosis exhibited lower GCS scores, higher blood lactate levels, lower fibrinogen levels, longer hospital stays, a higher incidence of pulmonary infection, a higher incidence of stress ulcers, and lower GOS scores. The GCS score upon admission is one of the rapid assessment tools for evaluating the condition of patients with traumatic brain injury, and a lower score indicates a more severe condition. On the other hand, blood lactate levels upon admission reflect both the metabolic perfusion status and serve as biomarkers for the body’s stress response. When the body is under stress, stress-induced hyperglycemia may occur [15]. Existing evidence suggests that in patients with moderate to severe traumatic brain injury, those who did not survive had higher blood lactate levels upon admission, indicating a correlation between elevated lactate levels, high blood glucose levels, abnormal coagulation function, and the severity of brain injury [16]. However, further research is needed to investigate the relationship between elevated blood lactate levels and stress-induced hyperglycemia in children with traumatic brain injury. Fibrinogen, coagulation factor I, participates in the process of thrombus formation through interactions with platelets and other coagulation factors. Animal model studies have shown that the consumption of fibrinogen can mitigate the progression and severity of certain diseases and may serve as a potential therapeutic approach [17]. In our study, 34 of the 80 patients showed decreased fibrinogen levels, which may indicate a poor prognosis in children with traumatic brain injury, consistent with the findings in Lv’s study [18]. Additionally, some researchers have studied the ratio of D-dimer to fibrinogen upon admission as a predictive factor for the progression of intracranial hematoma. A study from Japan confirmed that supplementing fresh frozen plasma to raise fibrinogen levels can improve the prognosis of patients with traumatic brain injury [19]. Some studies have indicated that elevated fibrinogen levels increase the risk of other complications, such as thrombosis [20, 21].

Honestly, most of the results are derived from retrospective studies, but there is a lack of evidence from large-scale, multicenter prospective studies. Therefore, for pediatric traumatic brain injury, the optimal fibrinogen level for improving prognosis still requires further analysis using large-scale data. Additionally, in our data analysis, we found that patients with elevated blood lactate levels had lower fibrinogen levels. Whether there is a correlation between elevated blood lactate levels, representative of coagulation function, and fibrinogen levels in pediatric traumatic brain injury needs to be further confirmed.

## Data Availability

All the data obtained and materials analyzed in this research are available with the corresponding author upon reasonable request.
